# Evaluation of Continuous Lactate Monitoring Systems within a Heparinized In Vivo Porcine Model Intravenously and Subcutaneously

**DOI:** 10.3390/bios8040122

**Published:** 2018-12-04

**Authors:** Alexander Wolf, Kevin Renehan, Kenneth K. Y. Ho, Benjamin D. Carr, Chieh V. Chen, Marie S. Cornell, Minyi Ye, Alvaro Rojas-Peña, Hao Chen

**Affiliations:** 1 Biocrede Inc., Plymouth, MI 48170, USA; alexander.wolf@biocrede.com (A.W.); kevin.renehan@biocrede.com (K.R.); kenneth.ho@biocrede.com (K.K.Y.H.); chieh.07@gmail.com (C.V.C.); miya.ye@biocrede.com (M.Y.); 2 Department of Surgery—ECLS Laboratory, University of Michigan Medical School, Ann Arbor, MI 48109, USA; bdecarr@umich.edu (B.D.C.); mhaeussl@med.umich.edu (M.S.C.); alvaror@umich.edu (A.R.-P.); 3 Section of Transplantation, University of Michigan Medical School, Ann Arbor, MI 48109, USA

**Keywords:** continuous blood lactate monitoring, lactate sensors, intravenous, subcutaneous, congenital heart disease, cardiopulmonary bypass surgeries

## Abstract

We present an animal model used to evaluate the in vivo performance of electrochemical amperometric continuous lactate sensors compared to blood gas instruments. Electrochemical lactate sensors were fabricated, placed into 5 Fr central venous catheters (CVCs), and paired with wireless potentiostat devices. Following in vivo evaluation and calibration, sensors were placed within the jugular and femoral veins of a porcine subject as a preliminary assessment of in vivo measurement accuracy. The mobile electronic circuit potentiostat devices supplied the operational voltage for the sensors, measured the resultant steady-state current, and recorded the sensor response values in internal memory storages. An in vivo time trace of implanted intravenous (IV) sensors demonstrated lactate values that correlated well with the discrete measurements of blood samples on a benchtop point-of-care sensor-based instrument. Currents measured continuously from the implanted lactate sensors over 10 h were converted into lactate concentration values through use of a two-point in vivo calibration. Study shows that intravenously implanted sensors had more accurate readings, faster peak-reaching rates, and shorter peak-detection times compared to subcutaneously placed sensors. IV implanted and subcutaneously placed sensors closer to the upper body (in this case neck) showed faster response rates and more accurate measurements compared to those implanted in the lower portion of the porcine model. This study represents an important milestone not only towards continuous lactate monitoring for early diagnosis and intervention in neonatal patients with congenital heart disease undergoing cardiopulmonary bypass surgeries, but also in the intervention of critical ill patients in the Intensive Care Units or during complex surgical procedures.

## 1. Background

The gold standard in the field of biomedical sensors is the development of intravascular/intravenous (IV) devices that can be used for real-time monitoring of blood pH, blood gases (*P*CO_2_, *P*O_2_), electrolytes (Na^+^, K^+^, Ca^2+^), and lactate and glucose. Importantly, close monitoring of lactate levels on a continuous basis dramatically changes the way critically ill patients are treated within intensive/critical care units (ICU) [1,2,3]. Hyperlactatemia, where patients have lactate levels of >2 mmol/L, has been shown to be a predictor of mortality in different groups of critically ill patients with sepsis, organ failure, trauma, or acute inflammatory response syndrome [4,5,6,7,8,9,10]. Further, lactate levels are being used to guide the management of early directed therapy for resuscitation of sepsis patients with lactate levels of >4 mmol/L [11,12,13]. Among the 1% of the 1.3 billion infants born globally each year that have congenital heart disease (CHD) [3], a quarter require interventions within the first year of life. In infants undergoing cardiopulmonary bypass (CPB) surgeries, 8.2% experience adverse outcomes and require Extracorporeal Membrane Oxygenation (ECMO) support (3.9%), dialysis (3.0%) due to kidney injury, and some face death (4.8%) [1,2]. If blood lactate levels of these infant patients were continuously monitored, abnormal lactate increase could be quickly detected and first-line treatments provided. Treatments include providing packed red blood cell (PRBC) transfusion to increase hemoglobin and O_2_ carrying capacity; providing systemic cooling, sedation, neuromuscular blockade to decrease oxygen consumption; and providing the institution of vasoactive-inotropic drugs such as milrinone to increase contractility and decrease systemic vascular resistance. Findings confirm that lactate monitoring is a valuable parameter in the early resuscitation of critically ill patients with organ failure and sepsis, and lactate-assisted therapy significantly reduced hospital mortality in patients with hyperlactatemia on ICU admission when lactate levels were decreased by >20% [4,14].

Despite extensive efforts over many years [15,16,17,18,19,20,21,22,23,24,25], significant lactate monitoring accuracy problems remain, and there are currently no IV chemical sensors (i.e., within the blood stream) commercially available for continuous real-time monitoring of ICU or surgical patients [26,27]. Typical assessment of a patient’s blood lactate concentration is carried out through drawing a sample via IV access line and running the sample through a gold standard benchtop blood gas analyzer to provide discrete measurements of multiple blood analytes (such as *P*O_2_, *P*CO_2_ pH, [Na^+^], [K^+^], [Ca^2+^]), and blood parameters (such as SO_2_, ctHbA, FO_2_Hb, FCOHb, FMetHb, and FHHb). A common observation for electrochemical sensors implanted in in vivo studies is a decaying performance pattern, where sensor output signals deviate significantly from those measured using blood gas machines over time, that is well-characterized, predictable, and reproducible. These IV sensor values for measured *P*O_2_ (and pH) are lower than the corresponding in vitro measurements, and similar errors are observed for glucose and lactate measurements [26]. This can be attributed to the adhesion of activated platelets forming blood clots on the surface of implanted sensors. Due to cellular respiration and consumption of oxygen/glucose, adhered platelets and other entrapped cells create a local environment that differs in *P*O_2_ and lactate/glucose levels compared to the bulk blood. Depending on the severity of thrombus formation, significant increase in sensor response time is also common and confound sensor output over time.

In this manuscript, we present preliminary results of in vivo continuous blood lactate measurements conducted by electrochemical enzyme sensors implanted within the jugular and femoral veins of a porcine model for 10 h, under anesthesia and systemic heparinization. Our solution is a combination of (1) a wire-type, immobilized enzyme based electrochemical lactate sensor mounted within (2) a polymeric, low-profile catheter tethered to (3) a wireless circuit module to monitor lactate levels, and store data. Antithrombotic properties of the catheter-housed sensor are achieved through the application of *S*-nitroso-N-acetylpenicillamine (SNAP) suspended within a silicone-based polymeric formulation. Upon sensor exposure to moisture and body temperatures, nitric oxide (NO) is released through this catheter housing, resulting in antimicrobial [28,29] and anti-platelet/anti-thrombotic activity that can prevent infection and thrombosis/fibrin sheath formation at sensor sites [30,31,32,33,34,35,36]. We use an in vivo healthy porcine model to test IV 5 Fr catheter sensors, designed to continuously measure blood lactate and lactate rates of change, to warn caregivers about imminent patient danger when abnormalities start to arise. 

## 2. Materials and Methods

### 2.1. Sample Fabrication, Preparation, NO Measurements

Lactate oxidase (from *Aerococcus viridans*), sodium L-lactate (sodium salt), glutaraldehyde, bovine serum albumin (BSA), fetal bovine serum (filtered, sterilized), phosphate buffered saline, iron (III) chloride (FeCl_3_), 37% hydrochloric acid (HCl), Nafion (5 wt% solution in lower aliphatic alcohols/H_2_O solution), 1,3-diaminobenzene, resorcinol, tetrahydrofuran (THF), poly(ethyleneimine) solution (PEI), glycerol, and Pluronic F-127 were all obtained from Sigma-Aldrich (St. Louis, MO, USA). Perfluoroalkoxy alkane (PFA)-coated platinum/iridium and silver wires are products of A-M Systems (Sequim, WA, USA). E2As Elast-Eon polyurethane was a gift from Aortech Biomaterials (Weybridge, Surrey, UK). 5-Fr central venous catheterization kits (Sungwon Medical, Korea), Bluetooth integrated potentiostat devices, in house made electrodes, device housings, and operational device firmware/hardware have been manufactured and assembled at Biocrede Inc. (Plymouth, MI, USA).

### 2.2. Sensor Fabrication and Assembly within Conduit

Lactate sensors were constructed based on previous designs [16,26,27,37,38] by first using a razor blade to cut a 1–2 mm length sensing region in the outer Teflon coating of the platinum/iridium wire (outer diameter = 0.2 mm) to function as the working electrode area. A thin film Nafion coating was applied to the working electrode surface, and then via a cyclic voltage (CV) electropolymerization process (cycling voltage between 0 and +0.830 V at 0.002 V s^−1^ for 18 h) a layer of polymerized resorcinol and *m*-phenylenediamine [39,40] was applied to the cavity. The polymerized layer is intended to promote rejection of electroactive interference species, such as ascorbic acid, uric acid, and acetaminophen, from reaching the working electrode region of the sensor surface which can cause interfering background current. A silver/silver chloride (Ag/AgCl) electrode composed of Teflon coated silver wire treated with acidified ferric chloride solution was tightly bound in close proximity to the working electrode sensing region to serve as the lactate sensor’s electrochemical reference. Heat-shrinkable polyester tubing was applied to secure the reference wire in place and mechanically reinforce the sensor element assembly. Lactate oxidase enzyme, stabilized within a PEI solution, was then immobilized within the cavity using glutaraldehyde. Once sufficient enzyme/glutaraldehyde crosslinking had formed, sensor outer layers consisting of a 5% (wt/vol) polyurethane and silicone rubber RTV in THF solution were top coated to restrict analyte diffusion to the enzyme layer. E2As polyurethane was previously used as a component of NO release polymeric coating to prevent platelet activation and thrombosis in a rabbit model undergoing extracorporeal circulation [41].

### 2.3. Sensor System and Animal Preparation

All protocols were approved by the University of Michigan Committee on Use and Care of Animals, and animals received humane care in accordance with the NIH Guide for the Care and Use of Laboratory Animals [42]. One adult healthy pig (42.5 kg, female) was sedated, anesthetized [43], and cut-downs were done on both groins and bilateral sides of the neck to isolate the femoral and jugular veins, and the right carotid artery. An arterial 14-gauge catheter was advanced into the right carotid artery to monitor systemic hemodynamics (arterial blood pressure and heart rate) and acquire blood samples for activated coagulation time (ACT) and in vitro arterial blood gas (ABG) values. The ACT and ABG were obtained using a Radiometer Medical ABL-800 standard blood gas analyzer (Copenhagen, Denmark) to establish baseline values. Mechanical ventilation settings were adjusted to maintain an end-tidal CO_2_ at 45 ± 5 mmHg and normal arterial blood gases. Three intravascular (venous) and four subcutaneous lactate sensors were placed in the animal (Table 1). 

**Figure 1:**
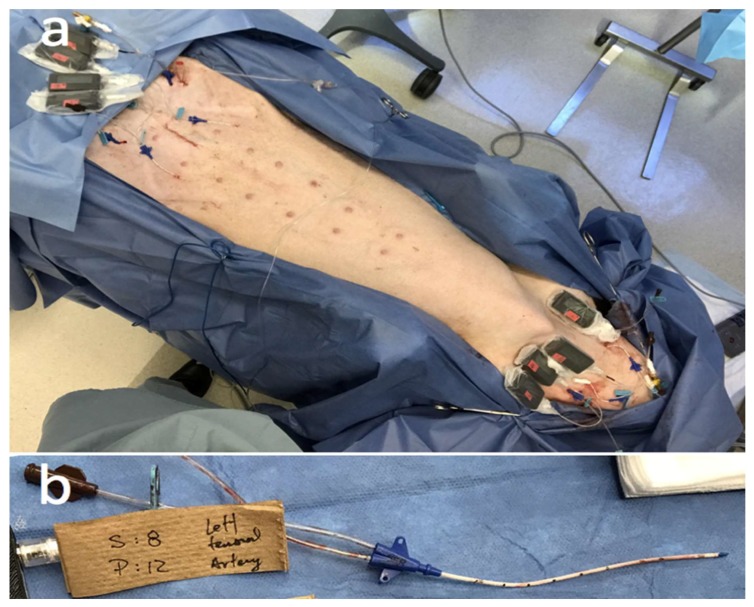
Intravenous and subcutaneous implantation of eight anti-thrombotic 5 Fr. Dual lumen central venous catheters lactate sensors in the animal study. (**a**) Catheter lactate sensors are placed in femoral arteries, jugular veins, and subcutaneous tissues of abdomen and neck in a porcine model. (**b**) An anti-thrombotic 5 Fr. Dual lumen central venous catheters lactate sensor after being carefully extracted upon excision.

After establishing the baseline for blood lactate and blood glucose via blood gas measurements, the IV lactate sensor systems were placed first in the femoral veins and subsequently in the jugular veins. Additional sensor systems were also placed into the subcutaneous tissues of the animal’s abdomen and neck, as this was determined to most closely approximate the environment of human abdominal subcutaneous tissue, where many continuous glucose sensor (CGM) systems are placed. The placements of lactate sensor systems are summarized in Table 1 and shown in Figure 1a. Note that one of the lactate sensors intended to be placed in the femoral vein was later found in the iliac vein upon excision of the catheter conduits at study end and was not included in the results section. A suprapubic Foley catheter was placed to monitor urine output (UOP).

ACT values of blood samples drawn from the carotid artery access line served as a metric for assessing the level of systemic anti-coagulation achieved through heparin bolus and adjusting the heparin continuous infusion rate. After the placement of all the sensor systems, a 250 U/Kg bolus of sodium heparin plus a heparin continuous infusion was initiated at a rate of 200 U/h. Target ACT’s for this study were 400–500 s (3–3.5× normal values), and heparin infusion was adjusted accordingly per heparin nomogram protocols. Arterial blood lactate remained stable during this time of heparin infusion adjustment and in vivo sensor acclimation. Calcium Gluconate, dextrose, Sodium Bicarbonate, Magnesium Sulfate, Potassium Chloride, THAM (TromeThamine injection), and crystalloids fluids were given to maintain normal electrolyte values.

### 2.4. In Vivo Experimental Interventions for Testing and Assessing Sensor Performance

Three methods were used to intervene the blood lactate and glucose level, as listed below:

1. *Intravenous Lactate Challenge:* Sodium L-lactate solution was infused intravenously. Sodium L-lactate (0.18 ± 0.02 moles) was dissolved in 20 mL sterile phosphate buffered saline (PBS), mixed thoroughly, and subsequently placed into an IV infusion bag with accompanying pump for administration a rate of 1500 mL/h over a 15-min infusion time.
2. *Intravenous Glucose Challenge:* 36 g, 18.0 mL bolus of 50% dextrose solution was given IV to the animal over 3 min.
3. *Respiratory Challenge:* Physiological lactic acidosis secondary to hypoxia and hypercapnia was induced by decreasing minute ventilation [44]. Hypercapnia/hypoxia was induced by first drawing blood from the animal and then infusing the blood back to the animal while decreasing 70–75% minute ventilation from baseline. Lower regions and tissues of the animal are distal from the heart and are responsible for circulation of blood and all corresponding analytes leading to lower perfusion efficiency compared to tissues proximal to the heart, accentuated during times of stress such as an induced respiratory challenge. It’s expected to see a signal response of greater magnitude and faster response time from the intravenous and subcutaneous sensors placed in the neck (proximal) than those sensor systems placed in the groin (distal).

**Figure 2:**
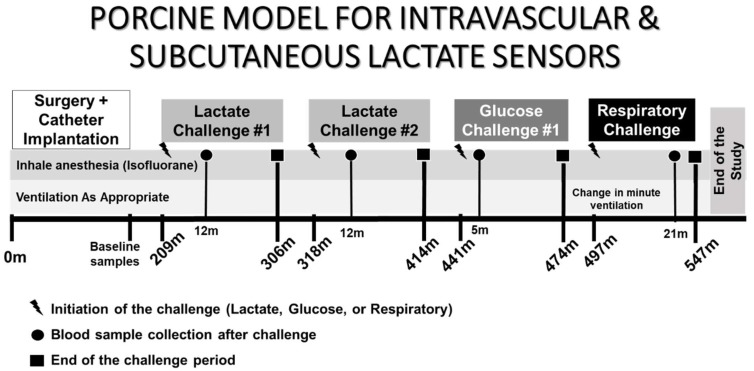
Timeline showing in vivo experimental interventions with the start and end of lactate, glucose, and respiratory challenges, along with the blood sample collection points.

The timeline for the initiation of intravenous lactate challenges, glucose challenge, and respiratory challenge is summarized in Figure 2. Briefly, the first intravenous lactate challenge was carried out at t = 209 min, followed by the second intravenous lactate challenge at t = 318 min. An intravenous glucose challenge was carried out at t = 441 min, and an induction of hypercapnia/hypoxia was carried out at t = 497 min. These were carried out far apart so that the blood lactate and glucose levels dropped back down to baseline level, as verified using a Radiometer Medical ABL-800 standard blood gas analyzer.

Two intravenous lactate challenges to induce an increase in blood lactate levels were used to verify that the sensors’ readings were comparable to those measured by the gold standard benchtop blood gas analyzer. Likewise, an intravenous glucose challenge to induce an increase in blood glucose levels was used to verify that the sensors’ lactate readings received no interference. Lastly, hypoxia and hypercapnia were induced to increase blood lactate and glucose levels to verify that the sensors responded correctly. 

## 3. Results

### 3.1. In Vivo Monitoring with Blood Lactate Modulation

After defining baseline lactate and glucose levels, two lactate challenges were performed as described earlier to obtain systemic lactate values of >7.0 mmol/L without affecting systemic glucose levels (Table 2).

After the first lactate infusion or challenge, blood lactate concentrations increased significantly (more than 10 times) compared to baseline without affecting systemic glucose levels. One hundred and five minutes after the initial infusion, systemic lactate values returned to normal and a second lactate challenge was performed (Table 2). During the first challenge, lactate values returned to normal levels of <2.0 mmol/L 90 min after an infusion to reach levels of 1.4 mmol/L.

Two hours after the second lactate challenge, a glucose challenge was performed. This bolus was not designed to provoke a sensor response but to identify any interference sensor response from non-specific enzymatic generation of hydrogen peroxide. Blood glucose concentrations increased by 40.0% compared to prior infusions without affecting systemic lactate levels (Table 2). The animal continued to clear both glucose and lactate from previous infusions.

To assess the sensitivity of the sensors to physiological lactic acidosis secondary to hypoxia and hypercapnia, a respiratory challenge was performed. Table 3 summarizes the changes observed during the respiratory challenge and shows how arterial blood gas trends describe a typical anaerobic metabolism, with signs of hypoxia and hypercapnia. 

**Figure 3:**
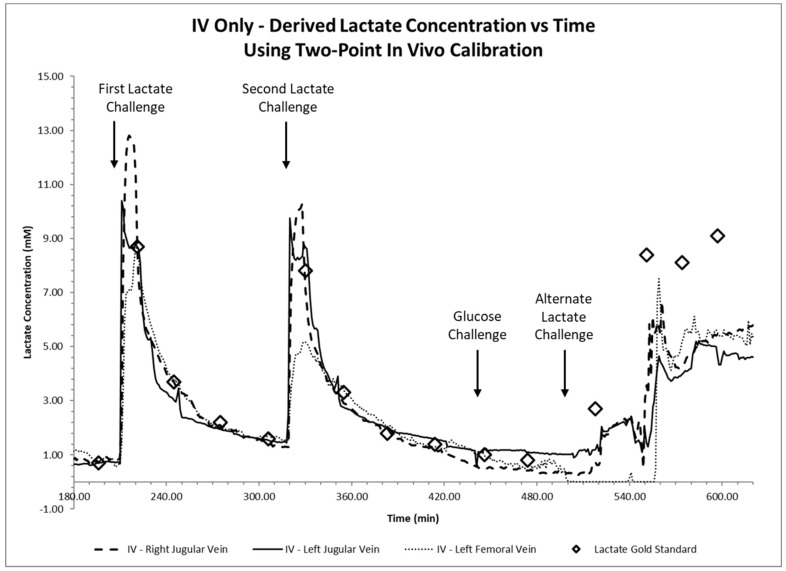
Continuous time trace of continuous intravenous (IV) lactate sensor measurements placed intravenously in the femoral or jugular veins are shown together with measurements using a blood gas machine (diamond markers) after lactate and glucose challenges (arrows).

**Figure 4:**
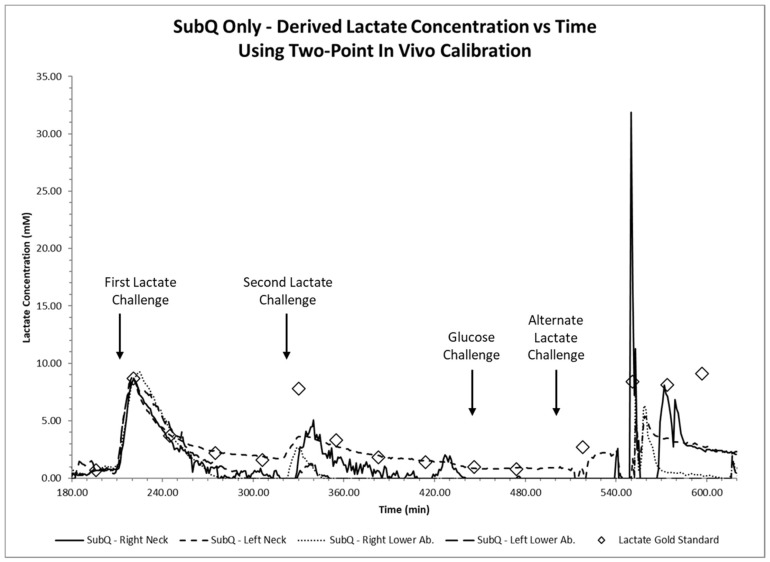
Continuous time trace of continuous subcutaneous (SubQ) lactate sensor measurements placed subcutaneously in the neck and lower abdomen shown together with measurements using a blood gas machine (diamond markers) after lactate and glucose challenges (arrows).

Lactate and glucose challenges, together with blood gas lactate concentration values taken from arterial blood samples were plotted as diamond markers and labeled in the figure legend as lactate gold standard values in Figure 3 and Figure 4. 

### 3.2. Intravenous and Subcutaneous Sensing Results

Recorded sensor output values were recorded as nanoamps (nA) and subsequently converted to mmol/L using a two-point conversion factor based on discrete lactate concentration values measured via the blood gas analyzer at 196 min and 221 min, before and after the first lactate infusion. Time traces of continuous intravenous lactate sensor measurements within the femoral and jugular veins of the porcine in vivo model are displayed in Figure 3, while time traces of continuous lactate sensor measurements placed subcutaneously near the neck and abdomen are displayed in Figure 4.

Sensor systems placed intravenously within the femoral and jugular veins of the porcine in vivo model showed sharp lactate increases followed by a gradual reduction upon both lactate challenges (Figure 3), showed minimal interference during the glucose challenge, and followed blood gas measurement values. We observe that sensor readings during the first lactate challenge showed readout deviations compared to those measured by the blood gas unit during the second lactate challenge (Figure 3). Sensors placed subcutaneously in the neck and lower abdomen tissues showed better lactate measurements during the first lactate challenge over the second challenge when compared to blood gas results and showed no significant interference after the glucose challenge (Figure 4). Intravenous sensor measurements near the neck showed superior performance.

## 4. Discussion

### 4.1. Intravenous and Subcutaneous Sensors: Response Times and Increase-Rates

Intravenously placed sensors showed steadier current measurements compared to those placed subcutaneously and responded to the first two lactate challenges within 3 min. Within these intravenously placed sensors, those placed in the upper body’s jugular vein responded much quicker compared to those placed in the lower body’s femoral vein (Figure 3). 

**Figure 5:**
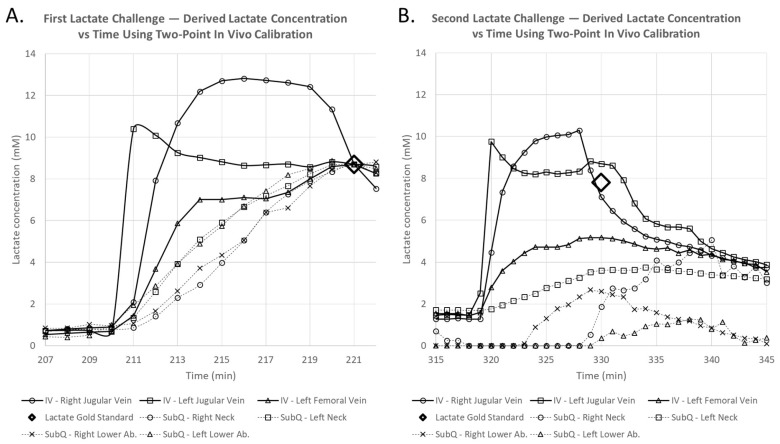
Detailed time traces of continuous intravenous (IV) and subcutaneous (SubQ) measurements of lactate sensors after (**A**) the first lactate challenge and (**B**) the second lactate challenge. Two lactate challenges were infused at 209 min and 318 min, and discrete blood gas measurements were performed at 221 min and 330 min from arterial blood samples taken via the carotid artery access line (diamond markers).

**Figure 6:**
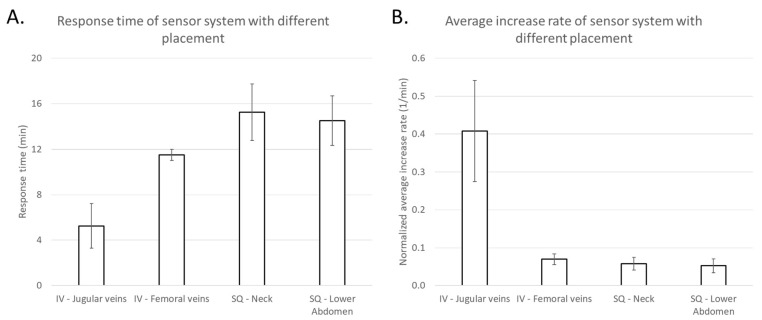
(**A**) Response times and (**B**) normalized average increase rates of sensors during both lactate challenges implanted intravenously in the jugular veins (*n* = 4) and femoral veins (*n* = 2), and subcutaneously in the neck (*n* = 4) and in the lower abdomen (*n* = 4). The error bar is defined as the standard error.

Intravenous and subcutaneous sensors’ lactate peak response times were identified after the first and second lactate challenges (Figure 5A,B) and both time-to-peak (response time) and average increase rates were calculated (Table 4) and plotted in Figure 6. Lactate sensors implanted subcutaneously showed longer response times of 11–22 min, compared to those implanted intravenously of 2–12 min. Importantly, sensors implanted intravenously in the jugular vein showed shorter response times of 2–10 min, compared to measurements from those implanted in the femoral vein of 11–12 min. Interestingly, response times of sensors placed subcutaneously in the upper body and in the lower body were similar and were 11–20 min. Average lactate increase rates were calculated (Table 4) and those sensors implanted intravenously in the jugular vein showed highest normalized rates of 0.145–0.669 min^−1^ compared to those implanted elsewhere with normalized rates of 0.010–0.095 min^−1^. Based on the limited sample size, the bootstrap 95% confidence intervals found for the response times and normalized average increase rates of sensors intravenously implanted in the jugular vein do not overlap with any of the corresponding confidence intervals for other sensors. This indicates significantly shorter response times and faster increase rates for sensors intravenously implanted in the jugular vein.

### 4.2. Intravenous and Subcutaneous Lactate Sensors: No Interference to Variations in Glucose

**Figure 7:**
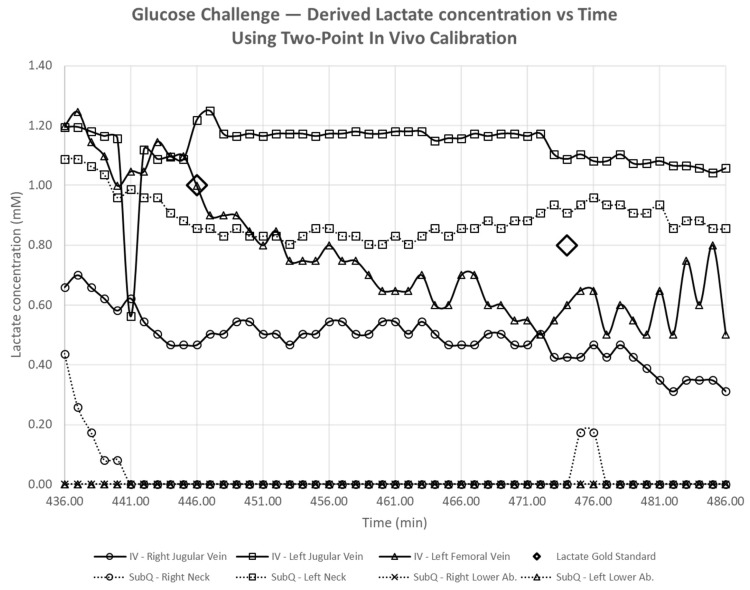
Continuous lactate measurements of implanted sensors during the glucose challenge. Dextrose was infused at 441 min, and discrete blood gas measurements were performed at 446 min and 474 min from arterial blood samples taken via the carotid artery access line (diamond marker).

An accurate in-line blood lactate sensor must possess high selectivity and receive minimal to no interference from all other analytes in the blood and thus we challenged the animal with glucose across all sensors as shown in Figure 3 and Figure 4, and sensors’ responses were analyzed as shown in Figure 7. Results show that lactate measurements from all subcutaneously and intravenously implanted sensors stayed flat throughout the glucose challenge compared to lactate levels measured by a blood gas analyzer at t = 446 min and t = 474 min. A Mann–Kendall statistical test was carried out for all the sensor readings from t = 436 min (5 min before the infusion of dextrose) to t = 486 min (45 min after the infusion of dextrose) and the results are summarized in Table 5. Note that the Mann–Kendall statistical test cannot be used for values very close to 0 and thus two subcutaneous sensors placed in lower abdomen were classified as N/A. The Kendall’s tau statistic values for all the other sensors were negative, as these showed a decreasing trend. Two-side *p*-values of all the other sensors except that of the subcutaneously implanted sensor in left neck were smaller than 0.05 confirming decreasing trends. Using the Mann–Kendall test we confirm non-significant glucose interference from enzymatic generation of hydrogen peroxide.

### 4.3. Intravenous and Subcutaneous Lactate Sensors: Accuracy and Performance

**Figure 8:**
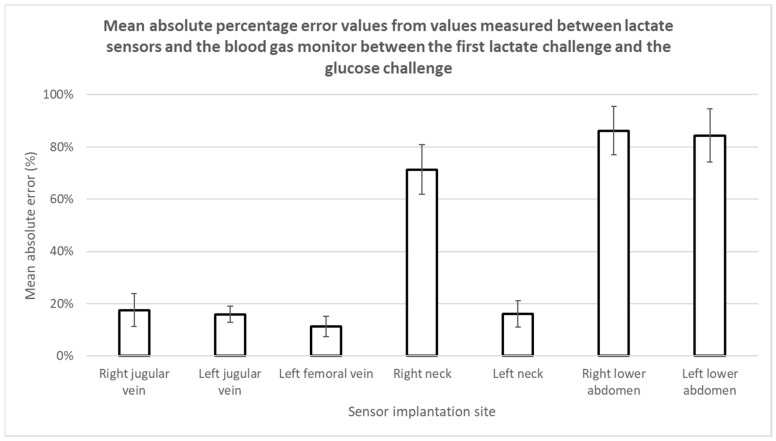
Mean absolute percentage errors calculated from the difference in measured lactate values performed between all implanted sensors and the blood gas analyzer across the first lactate and glucose challenges. Error bars describe the calculated standard errors for each sensor.

The accuracy and performance of the lactate sensors placed intravenously and subcutaneously were compared to measurements obtained from the blood gas analyzer. We theoretically label the differences in measured lactate values performed between the lactate sensors and the blood gas analyzer (considered the gold standard) as error. The absolute errors (%) of each sensor were computed from the beginning of the first lactate challenge to the end of the glucose challenge and are shown in Figure 8. Studies show that all intravenously implanted sensors have mean absolute percentage errors of 20% or smaller which is classified as clinically accurate [45] and are comparable or better to others [26]. On the other hand, only one subcutaneously implanted sensor had a mean absolute percentage error smaller than 20%, while the other three had mean absolute percentage errors of 70–90% as shown in Figure 4 and Figure 8. We suspect that such difference in performance could be a result from inadequate sensor polarization time or subcutaneous tissue thickness leading to much slower lactate diffusion rates from blood vessels. We ruled out sensor malfunction as these sensors were still tracking lactate changes after the respiratory challenge. Detailed explanations of the subcutaneously placed sensors are discussed in the next section.

Large discrepancies of lactate measurements performed between a sensor and the blood gas analyzer may indicate differing local lactate levels or poorly performing sensors. Poorly performing sensors can be attributed to assembly defects, incorrect in vivo placement, or possible but unlikely clot formation at the sensor site or vicinity of the sensing area. Further, the animal in this study encountered a phase of cardiac arrest at 550 min into the study due to irreversible ischemic injury to multiple organs and severe lactic acidosis secondary to a 1 h respiratory challenge. Thus, we suspect that the lactate measurements obtained after this event attributed to the higher deviation as shown in Figure 3 and Figure 4. Sensors placed in the jugular veins showed an increased blood lactate concentration following the respiratory challenge, while the sensors placed in the femoral veins did not (t = 500 min to t = 550 min). Note that all intravenously placed sensors continued to properly measure lactate concentrations of approximately 5 mM after t = 560 min (Figure 2), suggesting a local increase in blood lactate immediately after the start of the respiratory challenge. Note that in times of elevated stress, in this case a respiratory challenge, circulation is reduced in extremities and tissues located farther from the heart as part of a natural body survival mechanism, and it is likely that this event caused the discrepancy in sensor response of the lactate sensors placed in the femoral veins.

### 4.4. Subcutaneous Lactate Sensing Results Compared to Blood Lactate via Blood Gas

Lactate sensors were implanted in subcutaneous tissue to better understand the relationship between systemic blood lactate elevation through sodium lactate infusion challenges and elevated subcutaneous tissue lactate. There is a well-documented 5–15 min lag in response time between changes in blood glucose concentrations and changes in abdominal subcutaneous glucose, and this is a known drawback of commercial continuous glucose monitor (CGM) systems used for diabetes management [46,47,48,49]. However, the reduced invasiveness of subcutaneous tissue placement allows patients to insert or replace their sensors at home without medical assistance, which is extremely advantageous from a marketing perspective. Implanting lactate sensors subcutaneously and intravenously allows for data corroboration and to determine time delays and rate of change differences between the two. Since pigs and humans do not have the same abdominal subcutaneous or adipose tissue consistency, sensors were subcutaneously implanted near the neck and lower abdomen. Further, subcutaneous sensor implantation at this site reduced the probability of these being accidentally placed intramuscularly, while maintaining a similar environment compared to human abdomen near the stomach.

**Figure 9:**
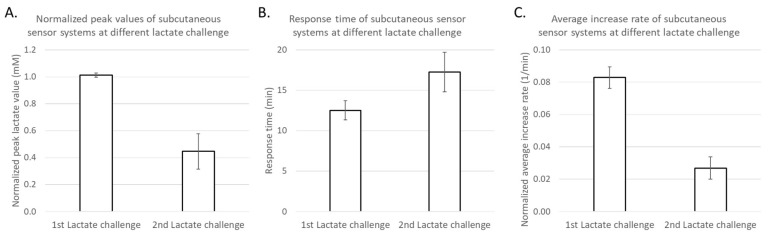
The (**A**) normalized peak lactate values, (**B**) response times, and (**C**) normalized average increase rates of subcutaneously implanted sensors during the first and second lactate challenges (*n* = 4 for each challenge). Error bars are described using standard errors.

Subcutaneously placed lactate sensors closely matched the blood gas arterial lactate values from time after placement and equilibration 4 h (240 min) into the study, as shown in Figure 4. Significant deviation from the gold standard was observed at 4.6 h (275 min), prior to the second lactate infusion challenge, leading to higher mean absolute percentage errors (Figure 8). All subcutaneously implanted sensors showed lower lactate concentrations than expected compared to blood gas measured values after the first lactate challenge, and sensors placed in the lower abdominal region showed lower values compared to those subcutaneously implanted near the neck. The intensity of normalized peak lactate values, response times, and normalized average increase rates of subcutaneously implanted sensors in the first and second lactate challenges were analyzed and presented in Figure 9. We use the bootstrap principle to show that subcutaneously implanted sensors have significantly lower peak lactate intensity and average increase rates in the second lactate challenge compared to the first one. 

Decreased sensor response indicated a reduced subcutaneous analyte diffusion to the sensing region or the active area of the sensor. This type of sensor performance may also be attributed to cell adhesion at the subcutaneous sensing region which creates an additional physical diffusion barrier for analytes to permeate through. On the other hand, if this type of sensor performance is seen in intravenously implanted sensors, it may be indicative of an obstructive clot preventing analyte exchange to the tissues or vessels surrounding the sensor which was not seen in our studies. Such performance can also result from inadequate time devoted to polarization of the sensor at the operational voltage or acclimating to the localized environment of the in vivo placement; however, this would not likely occur to several sensors simultaneously. Analyte exchange such as lactate levels between the blood in vessels and the subcutaneous tissues may have also provided reduced metabolic activity while the animal was under anesthesia, where decreased circulation to non-essential tissues/organs during prolonged anesthesia is a well-documented phenomenon [50]. We suspect that the subcutaneous tissues received inadequate circulation or perfusion due to the animal’s limited movement after the first lactate challenge not determinable using blood gas measurements. In this case, the subcutaneous sensors seemed to have experienced reduced signal intensity rather than abnormalities due to thrombosis or failing enzymatic activity. Further, subcutaneously implanted lactate sensors dropped to minimal levels during the second lactate challenge. 

### 4.5. Sensor Biocompatibility

**Figure 10:**
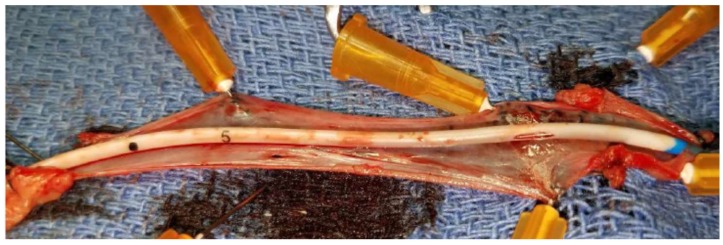
A NO releasing central venous catheter (CVC) with mounted electrochemical lactate sensor after 10 h of placement within a femoral vein during the in vivo porcine study. The catheter orifice provides a measurement window for the active sensing region.

At the conclusion of the in vivo study, the animal was euthanized 10 h after the implantation of lactate sensors for excision of the catheter conduits alongside the surrounding vessel. The presence of any thrombosis or cellular aggregation that could have impacted the proper flow of blood intravascularly or analyte diffusion via interstitial fluid subcutaneously to the active sensing regions were not observed as shown in Figure 1b and summarized in Table 1. Figure 10 displays the orifices of the lumens of catheter conduits containing the sensors placed intravenously in addition to the bisected blood vessel tissue which surrounded the catheter. Surface thrombosis and cellular aggregation were not expected for the subcutaneous sensors and thus not observed on the excised subcutaneous sensors. No visible clots were observed in the intact femoral or jugular veins before excision of the tissue, and no additional clotting or cell adhesion was observed in the surrounding venous tissue after excision and close examination. Thus, we assume that normal blood flow around the catheter or analyte diffusion to the sensor surface were not impacted. In addition, no significant thrombosis was observed on the catheter surface or at the sensing area of the catheter orifice, indicative of uneven, or irregular surface area. Since any presence of thrombosis or cellular aggregation around the sensors will affect the analyte diffusion around the sensing region and increase the sensor response time. We conclude that the absence of surface clotting indicated the retention of device accuracy, and thus assume that signal intensity and response time of the intravenous sensors were functioning normally throughout the study, as shown in Figure 3. 

### 4.6. Limitations of the Study

This study was limited by sensor production repeatability and the use of one single animal protocol (low sample size). Although this study suggested reproducible results with demonstrated feasibility after testing 7 sensors together at different implantation sites, a larger sample size might uncover potential technical complications. Although a high ACT anticoagulation regimen was used given the increased thrombogenicity of pig relative to humans, low hematoma formation or incontrollable bleeding was expected and none was observed. Lastly, prolonged anesthesia (10 h of inhaled anesthesia) associated with negative effects on systemic hemodynamics very likely was affected by the major decrease in minute ventilation. The animal developed hypoxia, hypercapnia, and irreversible multi-organ ischemia ending in cardiac arrest prior to sacrifice.

## 5. Conclusions

Based on the results of this in vivo heparinized porcine model study, the CVC lactate sensors fabricated and tested showed effective continuous lactate measurements (one per min). When calibrated and converted to lactate concentration values (mmol/L) via a two-point calibration, the lactate levels acquired intravenously in the jugular vein closely correlated with those measured by the blood gas analyzer. The lactate sensors followed periods of stable or baseline blood lactate levels, while peaks and elevated concentrations modulated through the application of sodium lactate infusions. These sensors were intended to quickly detect sharp increases in systemic blood lactate known to occur in patients and infants developing sepsis, complications with CHD, and extreme bodily duress. The lactate measurements from intravenous sensors accurately identified the start times of both lactate infusions with their respective decrease in lactate concentrations cleared by the kidneys, allowing the forecasting of alarming lactate increase rates within 2–15 min. Infusion challenges effectively modulated the animal’s arterial blood lactate levels in a dose-dependent response from baseline to elevated concentrations typically observed in neonatal critical ill patients. 

The sensors placed intravenously in the upper body’s jugular vein closely correlated with the discrete gold standard arterial blood gas lactate concentrations, compared to those sensors placed intravascularly in the lower body’s femoral vein, especially during the respiratory challenge leading to reduced circulation. Intravenously placed sensors displayed more accurate readings than those of subcutaneously placed sensors when all response times were compared to the discrete gold blood gas measurements. Namely, intravenous lactate and subcutaneous lactate peak-reaching rates and peak-detection times were measured to be 0.084–0.669 min^−1^ and 2–12 min compared to 0.010–0.095 min^−1^ and 11–22 min, respectively. Subcutaneously placed sensors displayed significantly longer sensor response times and reduced magnitudes during the periods of elevated systemic lactate concentration due to poor circulation, varying lactate diffusion rates through different tissues, and/or the inactivity of subcutaneous tissues in an anesthetized or otherwise immobilized animal. This in vivo porcine study evaluated the analytical performance of continuous NO releasing 5 Fr dual lumen lactate sensing systems to reduce/eliminate the occurrence of thrombosis at the sensor sites. Study results represent an important milestone to achieve continuous lactate monitoring for early diagnosis and intervention to avoid fatalities in critical ill patients or those undergoing CPB surgeries.

Future animal studies will be conducted intravenously in non-heparinized animals to evaluate the combined analytical accuracy with the anti-thrombotic properties incorporated into the NO-releasing sensor designs. Additional lactate infusion challenges, possibly in greater frequency per study or in greater magnitude/concentration to further establish the sensor system’s operational range and capabilities will be performed. Future designs will include display and data collection options for multiple simultaneous sensors as recommended by medical doctors.

## Figures and Tables

**Table 1:** Catheter sensor placement within the porcine model intravenously (IV) or subcutaneously (SQ), and device measurement and clotting performance.

Parameter	IV1	IV2	IV3	SQ1	SQ2	SQ3	SQ4
**Placement Location**	Right Jugular vein	Left Jugular vein	Left Femoral Vein	Right Neck	Left Neck	Right Lower Abdomen	Left Lower Abdomen
**Tissue Environment**	IV	IV	IV	SQ	SQ	SQ	SQ
**In Vivo Performance**	Close to Gold Standard	Close to Gold Standard	Close to Gold Standard, but reduced peak intensity	Significant deviation after 1st lactate challenge	Closest subcutaneous to Gold Standard, reduced peak intensity	Significant deviation after 1st lactate challenge	Significant deviation after 1st lactate challenge
**Clotting Observed**	NEGATIVE	NEGATIVE	NEGATIVE	NEGATIVE	NEGATIVE	NEGATIVE	NEGATIVE

**Table 2:** Summary of systemic arterial blood samples pre- and post-lactate and glucose challenges.

	Lactate Concentration (mmol/L)				Glucose Concentration (mg/dL)		
Lactate Challenge #	Initial	Post-infusion	25 min Post-infusion	60 min Post-infusion	Initial	25 min Post-infusion	60 min Post-infusion
1	0.7	8.9	3.7	2.2	102	114	130
2	1.6	7.8	3.3	1.8	136	131	134
	**Glucose Concentration (mg/dL)**				**Lactate Concentration (mmol/L)**		
Glucose Challenge #	Initial	5 min Post-infusion		30 min Post-infusion	Initial	5 min Post-infusion	30 min Post-infusion
1	156	218		175	1.4	1.0	0.8

**Table 3:** Respiratory challenge.

	PaCO_2_ (mmHg)	PaO_2_ (mmHg)	pH	SO_2_ %	Lactate (mmol/L)	Glucose (g/dL)
Initial	40.9	252	7.509	99.9	0.8	175
45 min Post-challenge	70.2	29.4	7.339	37.7	2.7	176

**Table 4:** Table describes times-to-peak for both infusion challenges with their respective normalized average rates of increase as measured by intravenously (IV) and subcutaneously (SQ) implanted sensors.

	IV—Right Jugular Vein	IV—Left Jugular Vein	IV—Left Femoral Vein	SQ—Right Neck	SQ—Left Neck	SQ—Right Lower Abdomen	SQ—Left Lower Abdomen
Time from 1st lactate infusion to peak (min)	7	2	12	12	11	16	11
Normalized average increase rate (1/min)	0.213	0.605	0.084	0.082	0.090	0.064	0.095
Time from 2nd lactate infusion to peak (min)	10	2	11	22	16	11	20
Normalized average increase rate (1/min)	0.145	0.669	0.055	0.037	0.021	0.039	0.010

**Table 5:** Mann–Kendall statistical test results for all implanted lactate sensors across t = 436 min (5 min before dextrose infusion) and t = 486 min (45 min after dextrose infusion).

	IV—Right Jugular Vein	IV—Left Jugular Vein	IV—Left Femoral Vein	SQ—Right Neck	SQ—Left Neck	SQ—Right Lower Abdomen	SQ—Left Lower Abdomen
Kendall’s tau	−0.674	−0.427	−0.745	−0.281	−0.0436	N/A	N/A
Two-sided *p*-value	5.60 × 10^−11^	2.50 × 10^−5^	7.60 × 10^−14^	0.0141	0.6744	N/A	N/A
